# Micron-sized biogenic and synthetic hollow mineral spheres occlude additives within single crystals

**DOI:** 10.1039/d1fd00095k

**Published:** 2021-12-08

**Authors:** Bartosz Marzec, Jessica Walker, Yasmeen Jhons, Fiona C. Meldrum, Michael Shaver, Fabio Nudelman

**Affiliations:** EaStCHEM School of Chemistry, University of Edinburgh Joseph Black Building, The King’s Buildings, David Brewster Road Edinburgh EH9 3FJ UK Fabio.nudelman@ed.ac.uk; JEOL UK Ltd 1-2 Silver Court, Watchmead Welwyn Garden City AL7 1LT UK; Beamline I14, Diamond Light Source, Harwell Science and Innovation Campus Didcot Oxfordshire OX11 0DE UK; School of Chemistry, University of Leeds Woodhouse Lane Leeds LS2 9JT UK; Department of Materials, School of Natural Sciences, The University of Manchester UK

## Abstract

Incorporating additives within host single crystals is an effective strategy for producing composite materials with tunable mechanical, magnetic and optical properties. The type of guest materials that can be occluded can be limited, however, as incorporation is a complex process depending on many factors including binding of the additive to the crystal surface, the rate of crystal growth and the stability of the additives in the crystallisation solution. In particular, the size of occluded guests has been restricted to a few angstroms – as for single molecules – to a few hundred nanometers – as for polymer vesicles and particles. Here, we present a synthetic approach for occluding micrometer-scale objects, including high-complexity unicellular organisms and synthetic hollow calcite spheres within calcite single crystals. Both of these objects can transport functional additives, including organic molecules and nanoparticles that would not otherwise occlude within calcite. Therefore, this method constitutes a generic approach using calcite as a delivery system for active compounds, while providing them with effective protection against environmental factors that could cause degradation.

## Abstract 2

Occlusion of micron-sized algae cells and calcitic hollow spheres within calcite single crystals, mediated by the positively charged polymer poly(allylamine hydrochloride). Both objects are used to transport functional additives to the host lattice.

## Graphical abstract

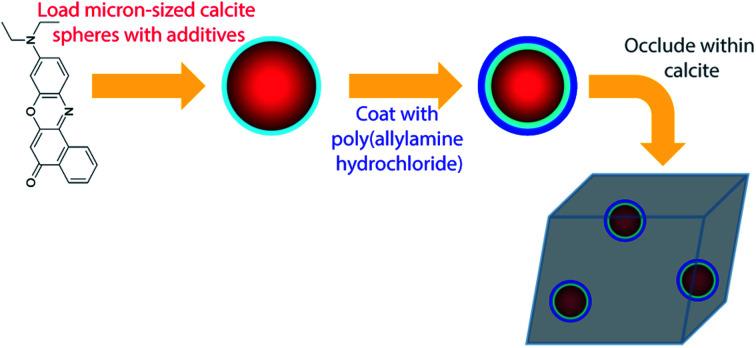

## Introduction

The formation of mineralized tissues is a widespread phenomenon in nature. Organisms from all 5 kingdoms are known to precipitate more than 60 different types of mineral, each tailor-made for their biological functions.^1^ Examples include vertebrate bone and teeth, mollusc shells, sea urchin spines, silica spicules in sponges and magnetite and greigite particles found in magnetotactic bacteria. The formation of mineralized tissues is a tightly regulated process, where a water-insoluble organic matrix provides a three-dimensional framework and scaffold in which the mineral forms,^1^ and water-soluble proteins and polysaccharides that participate in crystal nucleation and growth.^2–4^ These additives can also become incorporated within the crystal, modifying mechanical properties.^5,6^

The ability of water-soluble macromolecules to be incorporated within crystalline hosts and to modify their morphologies and material properties has been the focus of significant attention over recent years, both in biomineralization and in bio-inspired crystallization studies.^7^ It has led to a variety of synthetic approaches for the incorporation of different guest species within host crystals.^5,8–15^ Calcium carbonate, and in particular the thermodynamically stable polymorph calcite, has been widely used as a model system in such studies. A range of substances, including small molecules,^8,15–17^ nanoparticles,^11,18^ carbon nanotubes,^19^ gel networks,^20,21^ polymer nano-objects,^5,22–24^ and beads,^25^ emulsions^26^ and protein nanogels^27^ have been incorporated within single crystals of calcite and other minerals.^11,28,29^ This has led to the manufacture of single-crystal composites with unique optical, mechanical and magnetic properties.^11,13,30–33^ Occlusion of micron-sized species within calcite in the form of *Escherichia coli*^34^ and the algae *Chlorella* sp.^35^ cells, on the other hand, has been met with limited success. In both cases, the cells were confined to the surface of the crystal and only partially occluded. This shows that there are still limitations in terms of the size of the guest species that can be incorporated within crystals. While additives ranging from single molecules to a few hundreds of nanometers in size can be occluded, the incorporation of larger, micron-sized guests is still challenging.

In order to be occluded within a single crystal, additives must bind to the crystal surface such that they are resident long enough to be incorporated, but do not strongly inhibit crystal growth. The primary strategy used to engineer incorporation has been functionalising particles with block copolymers rich in anionic groups including carboxylates, sulfates, sulfonates, phosphates and phosphonates.^9,10,14,36–38^ Notably, nanoparticles functionalised with low charge hydroxyl-rich groups incorporate at very high levels due to their strong binding to the crystal surface and high colloidal stability in the crystallisation solution.^11^ The latter enables high concentrations of particles to be employed. Control over parameters such as the nature of the functional groups, their density and chain length, therefore offers a basis for optimizing the incorporation of particles inside single crystals.

**Fig. 1:**
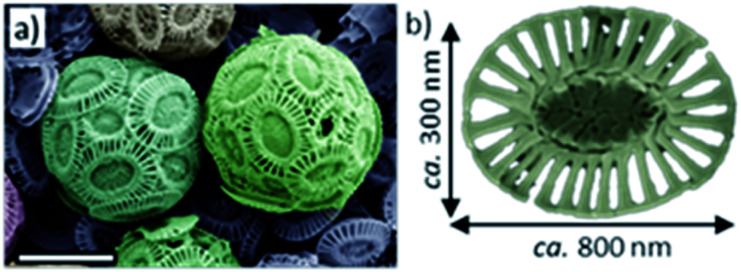
(a) False-colored SEM image of *Emiliania huxleyi* coccospheres isolated from their growth medium. (b) An individual coccolith.

Incorporation within single crystals also offers an attractive means of protecting active compounds, where they are completely isolated from the environment within the impermeable crystal. This strategy has been demonstrated previously by occluding protein nanogels loaded with active compounds within calcite.^27^ Here, we explore the potential of achieving much higher loading levels by occluding micron-sized structures within calcite single crystals. Two systems are investigated: in the first, 2 μm large cells of the calcifying unicellular marine alga *Emiliania huxleyi* (‘Ehux’) are entrapped. These cells produce coccoliths, which are disk-shaped assemblies of calcite single crystals that form an exoskeleton around the cells, termed the coccosphere (Fig. 1). In addition to the cytoskeleton and biomolecules normally present within any cell, Ehux can be pre-loaded with a variety of additives, including fluorescent molecules and inorganic nanoparticles, and can transport them into calcite hosts to produce single-crystalline functional composites. In the second system, this methodology is extended to synthetic hollow calcite spheres, offering the ability to deliver large synthetic and biological structures occluded within single crystals. Notably, incorporation is achieved by functionalizing both types of additives with commercially-available poly(allylamine hydrochloride) (PAH). While additives used to direct calcium carbonate precipitation are usually anionic,^9,12,13,26,38^ this work demonstrates the potential of using cationic additives to drive effective occlusion.

## Results

### Occlusion of *E. huxleyi* cells in calcite

Ehux cells were isolated from their growth medium (see Experimental) and re-dispersed in a supersaturated CaCO_3_ growth mixture obtained by mixing equal volumes of 20 mM CaCl_2_ and 20 mM NaHCO_3_, and containing 10 μM of PAH. This polymer is positively-charged under these reaction conditions (≈pH 9) and has been previously shown to modify calcium carbonate precipitation, leading to the formation of thin films and fibers of calcite particles.^39^ At specific time points (5 min, 20 min, 60 min, 24 h, 48 h and 96 h), samples were removed from the crystallization medium and imaged using scanning electron microscopy (SEM).

**Fig. 2:**
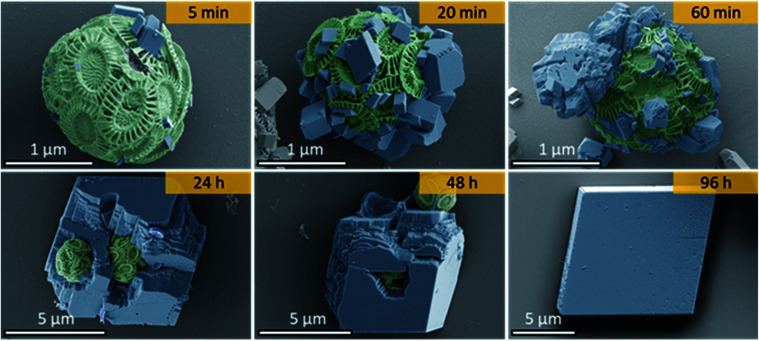
Time-resolved false colored SEM images showing the occlusion of Ehux coccospheres within calcite single crystals. For clarity, the coccospheres have been highlighted green and the freshly deposited calcite units have been colored blue. Each image was taken from a different sample, collected at different time points.

**Fig. 3:**
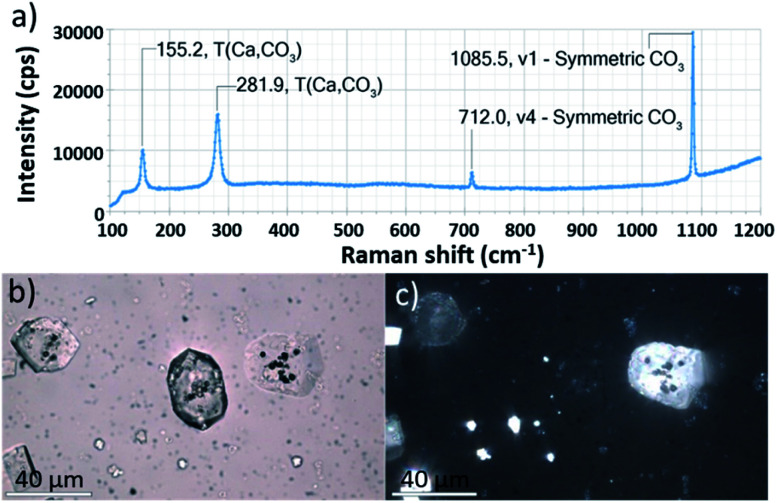
(a) Raman spectroscopy of a crystal containing Ehux occlusion. (b) Bright field and (c) corresponding crossed-polarized image of calcite crystals containing Ehux occlusions recorded using a petrographic microscope. Scale bars: 40 μm.

As shown in Fig. 2, calcite crystals nucleated on the surface of the PAH-coated coccospheres. A few crystals were present after 5 min, and were more abundant after 20 and 60 min. The structure then developed such that a single crystal surrounded the cells after 24 h and 48 h, and the cells were completely encapsulated inside a single crystal after 96 h. The resulting particles were 10–20 μm in size and displayed well-defined rhombohedral morphologies. Raman spectroscopy confirmed that the crystals containing the Ehux cells were calcite (Fig. 3a), and polarized light microscopy showed that they were completely birefringent when viewed with crossed-polarizers (Fig. 3b and c).

**Fig. 4:**
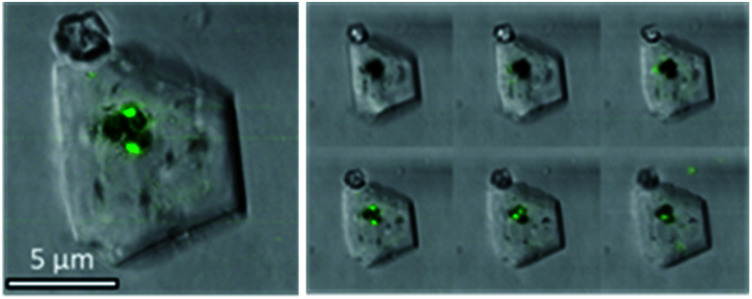
Confocal microscopy image of a representative calcite crystal containing Ehux cells with the overlay of bright field and fluorescence. The right panel shows images of the crystal obtained at different focal planes. (*λ*_e*x*_ = 458 nm, *λ*_em_ = 630–660 nm).

To further confirm that the Ehux cells were entrapped within individual calcite crystals, occluded, the composite crystals were imaged using confocal laser scanning microscopy (CLSM) based on the detection of chlorophyll autofluorescence. Fluorescence was detected within the calcite (Fig. 4, left), in the focal planes that passed through the middle crystals (Fig. 4, right). This not only confirmed that the cells were indeed located within the calcite crystals, but also demonstrated that the aqueous interior of the cells were retained during crystallization.

**Fig. 5:**
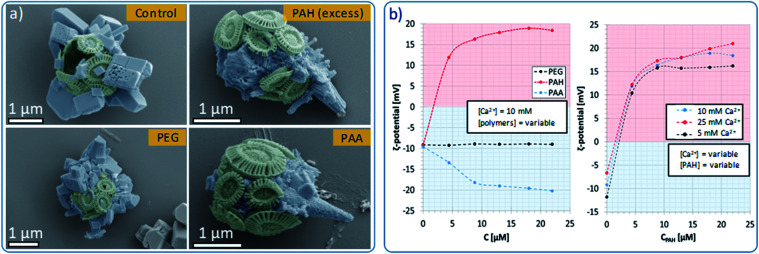
Effects of polymeric habit modifiers on the morphology and *ζ*-potential of Ehux coccospheres. (a) False color SEM images of coccospheres placed in supersaturated CaCO_3_ solutions containing no additives (control), or synthetic polymers, recorded after 96 hours. (b) *ζ*-Potential measurements recorded for Ehux coccospheres dispersed in 10 mM CaCl_2_ containing variable concentrations of PAH, PEG and PAA (left), and coccospheres dispersed in mixtures containing various concentrations of CaCl_2_ and different amounts of PAH (right).

Control experiments were also conducted in the absence of PAH and using different organic additives (Fig. 5a). With no PAH present, multiple well-defined calcite rhombohedra formed on the exterior of the coccosphere. These crystallites were randomly oriented. PAH concentrations above 15 μM resulted in the formation of a calcium carbonate film over the surface of the coccoliths, together with some rod- or fiber-shaped protrusions. Both thin films and fibers are characteristic of a polymer-induced liquid precursor (‘PILP’) phase,^40^ which is known to form in the presence of PAH.^41^ Experiments were carried out using polymers with different charges: neutral poly(ethylene oxide) (PEG) and negatively charged poly(acrylic acid) (PAA). As shown, 10 μM PEG had little effect on calcite formation, leading to the formation of *ca.* 1 μm calcite crystals on the coccospheres’ surfaces. In contrast, addition of 10 μM PAA resulted in the deposition of rod-shaped mineral protrusions after 96 h.

### Interaction of PAH with the coccospheres

To further probe the interaction between the polymers and the surface of the cells, we measured the *ζ*-potential of the coccospheres under different experimental conditions. As shown in Fig. 5b (left), coccoliths dispersed in CaCl_2_ had a *ζ*-potential of −10 mV, which increased to + 12 mV upon addition of 5 μM of PAH, and + 16 mV on addition of 10 μM of PAH. This increase in the *ζ*-potential confirms that PAH binds to the surface of the cells, likely mediated *via* electrostatic interactions with the negatively charged coccospheres.

**Fig. 6:**
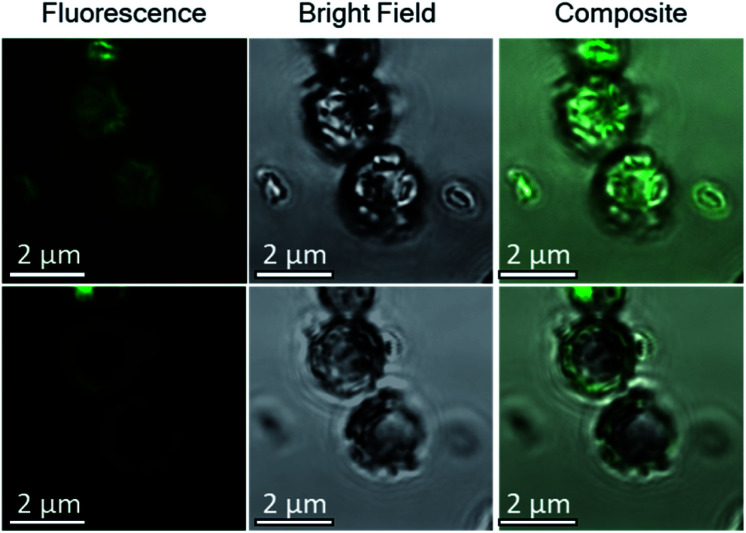
Confocal microscopy images of Ehux cells dispersed in a growth mixture containing fluorescently-labelled PAH, recorded at two different focal planes: at 0 μm (top row), corresponding to the top surface of the coccospheres, and at −1 μm (bottom row), which corresponds to a cross-section through the coccosphere and the cells. Left column: fluorescence image. Middle column: bright field image. Right column: overlay of fluorescence and bright field images.

CLSM imaging of cells treated with PAH labelled with a fluorescent substituent further confirmed the adsorption of the PAH. Images taken at the focal plane corresponding to the surface of the algae (Fig. 6, top row) show fluorescent coccoliths. In images taken at the focal plane cutting through a cross-section of the cells (bottom row), the fluorescence signal was visible as a green ring surrounding dark cells, showing that the polymer was located on the coccosphere and absent from the interior of the algae.

Concentrations of PAH above 10 μM only resulted in small increases in the *ζ*-potential, reaching a plateau close to + 18 mV at concentrations of 13 μM and above (Fig. 5b). This shows that above [PAH] = 10 μM, the coccospheres became saturated with the polymer and the excess remains in the crystallization solution, where it modifies the formation of the calcite crystals, leading to the formation of fibrous structures (Fig. 5a). The interaction between PAH and the coccospheres was independent of the CaCl_2_ concentration as the *ζ*-potential followed a similar trend and reached similar values when [Ca^2+^] = 5, 10 and 25 mM (Fig. 5b, right).

As presented in Fig. 5b (left), neutral PEG did not affect the *ζ*-potential of the coccospheres, and the negatively charged PAA induced a change from −10 mV to −20 mV due to binding to the surface of the coccoliths. Given that both the polymer and the coccospheres are negatively charged, it is conceivable that such binding is mediated by calcium ions. In common with PAH, the negative surface charge reached a plateau at polymer concentrations above 10 μM, suggesting that the cells become saturated with PAA and that the excess remains in the solution.

### Use of *E. huxleyi* cells to occlude fluorescent dyes within calcite

**Fig. 7:**
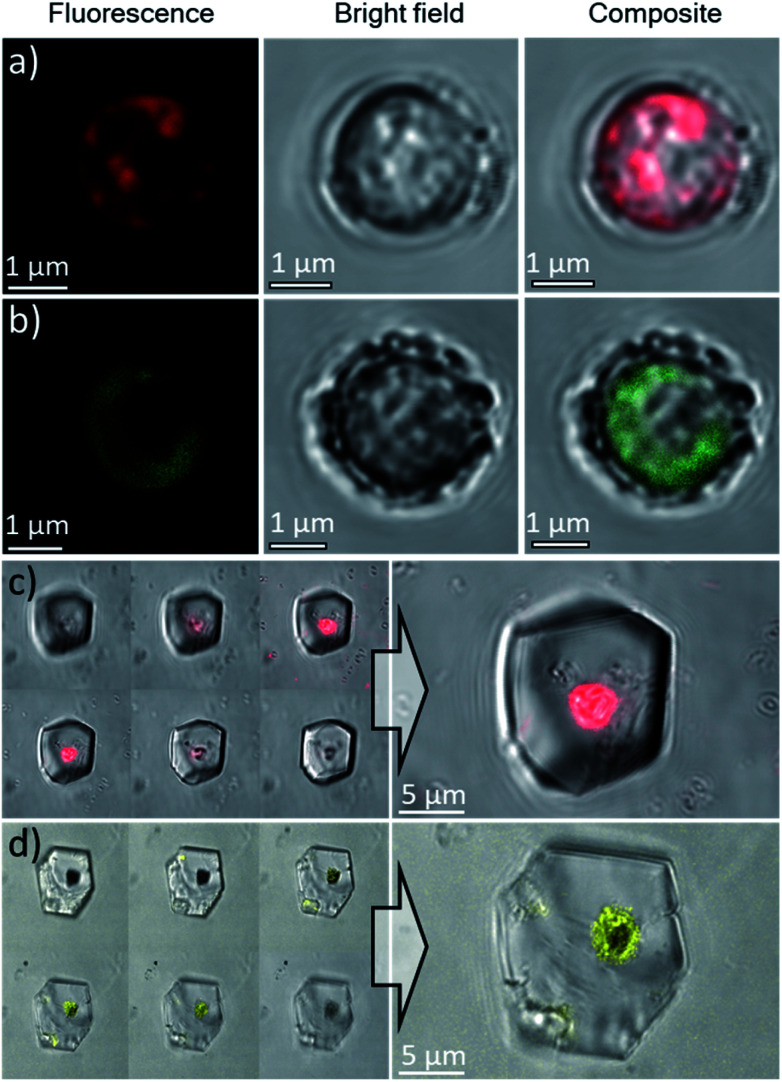
Confocal microscopy images of Ehux cells pre-loaded with: (a) Nile Red, *λ*_ex_ = 514 nm, *λ*_em_ = 595–625 nm and (b) CdTe quantum dots, *λ*_ex_ = 458 nm, *λ*_em_ = 495–515 nm. Scale bars: 1 μm. Confocal microscopy images of calcite crystals containing Ehux cells pre-loaded with: (c) Nile Red and (d) CdTe quantum dots, inside calcite crystals. The images are the overlay of fluorescence and bright field images. Left panel: images at different focal planes.

Based on these optimized occlusion conditions, the possibility of using the algae as delivery vehicles for non-biological materials was then explored. Ehux cells were cultured in the presence of a mixture of Nile Red (an organic fluorescent probe used to highlight cellular membranes)^42^ and Triton-X100 (a surfactant disrupting cellular membranes and facilitating additives uptake), or CdTe quantum dots decorated with carboxylate functionalities, where these exhibit well-defined fluorescence.^43^ The successful uptake of these materials was confirmed with CLSM, where both compounds were imaged inside the cells (Fig. 7a and b). Cells pre-loaded with the synthetic fluorescent probes were then used in a CaCO_3_ crystallization experiment, resulting in calcite single crystals containing cellular occlusions. As shown in Fig. 7c and d, fluorescence images taken at different focal planes showed that fluorescence originating from the synthetic probes was detected inside the crystals (Fig. 7c and d, left panel), demonstrating that the unicellular organisms can be used as carriers to occlude additives within single crystalline calcite.

### Occlusion of polycrystalline hollow spheres of CaCO_3_ in single crystalline calcite

**Fig. 8:**
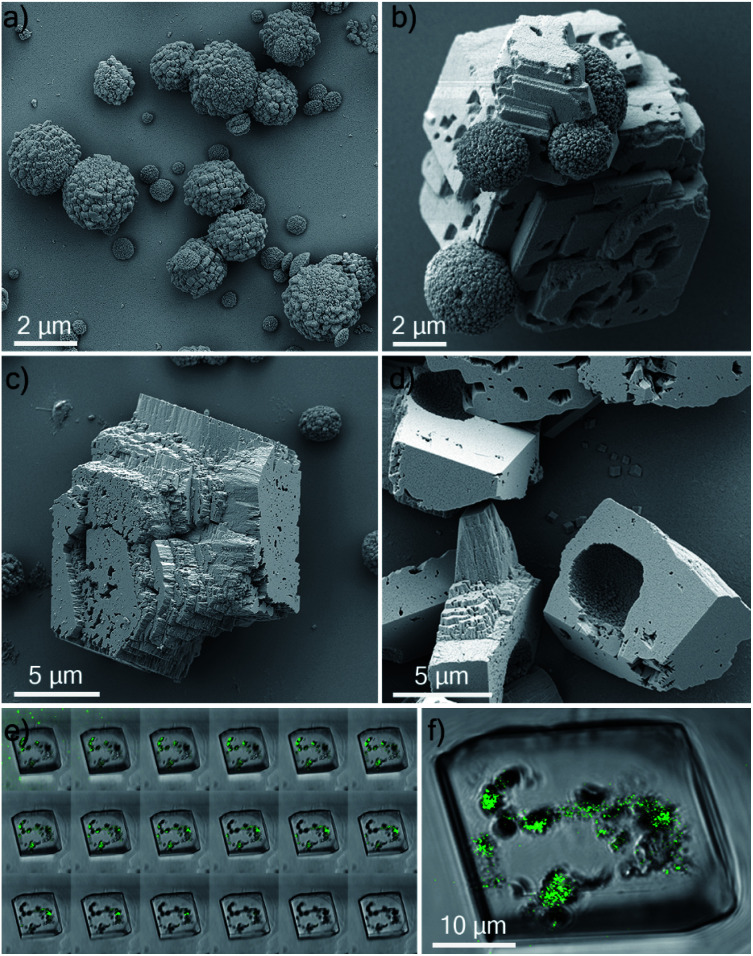
(a) SEM image of synthetic hollow calcite spheres. (b) SEM image of a growing calcite crystal incorporating calcite spheres. (c) SEM image of a mature calcite crystal where occluding calcite spheres are no longer visible. (d) SEM image of calcite crystals crushed between two glass slides, revealing hollow imprints whose diameters correspond to the diameter of the occluded calcite capsules. Confocal microscopy images of a calcite single crystal containing occlusions of synthetic hollow spheres pre-loaded with an aqueous solution of HPTS (*λ*_ex_ = 448 nm, *λ*_em_ = 490–510 nm): (e) *Z*-stack of the crystal with images taken at different focal planes and (f) representative image of the crystal. The images are the overlay of fluorescence and bright field.

Having demonstrated the successful occlusion of coccolithophores using PAH, we then extended our strategy to synthetic polycrystalline hollow calcite capsules. These were 2–4 μm in diameter (Fig. 8a) and were precipitated from a water/hexane mixture using the method of Fujiwara *et al.*^44^ These behaved similarly to the coccospheres in seeds in CaCO_3_ crystallization experiments in that they were only occluded within calcite crystals when PAH was present (Fig. 8b and c). Their occlusion was confirmed by fracturing the composite crystals, where SEM analysis revealed the presence of regular, spherical imprints of similar diameter as the original calcite hollow spheres (Fig. 8d). These synthetic capsules were also explored as delivery vehicles, and were pre-loaded with an aqueous solution of a fluorescent probe 8-hydroxy-1,3,6-trisulfonic acid (HPTS). As shown in Fig. 8e and f, fluorescent calcite spheres were confirmed to be present with the calcite hosts. This system therefore provides a versatile and simple strategy for encapsulating large solution volumes – containing selected payloads – within single crystals.

## Discussion

A wide range of guest species ranging from small molecules to nanoparticles, gels and submicron particles have been occluded within calcite single crystals. However, while it is straightforward to incorporate low concentrations of additives, achieving high loading levels has proven far more challenging. This has driven efforts to determine “design-rules” that govern successful incorporation, where this is clearly a complex process determined by multiple factors.

In order for additives in this size range to be incorporated it is essential that they are present on the crystal surface when the growth steps propagate. The coverage of additives on the surface is therefore determined by the concentration of additives present in the solution and their residence time on the surface, where higher concentrations and binding strengths will drive higher occlusion. However, it is not as simple as just increasing binding strength as strongly-binding additives also inhibit crystal growth, often leading to the formation of polycrystalline particles and sometimes a change in polymorph. Similarly, while it may appear straightforward to just increase the concentration of additive in the crystallisation solution, this can often lead to aggregation – particularly for the anionic additives that bind strongly to calcite. This ultimately reduces the concentration of free particles available to bind to the crystal surface.

A “sweet-spot” therefore exists in the binding strength of the additive to the crystal surface, and this has been seen in a number of systems. Gold nanoparticles functionalised with low charge, hydroxyl-rich proteins or synthetic polymers incorporated within calcite at exceptionally high levels, where these particles bind effectively and are extremely stable in the crystallisation solution.^11^ A series of block copolymer micelles were also synthesized that exhibited different ratios of surface chains with either carboxylate (negatively charged) or hydroxyl (neutral) functional groups.^12^ The occlusion efficiency did not directly scale with the carboxylate content of the steric stabilizers, where nanoparticles comprising 1 : 1 carboxylate/hydroxyl groups incorporated at higher levels than those that only possessed carboxylate chains. Adding further to the degree of complexity, occlusion also depends on the rate of crystal growth. Weakly-binding additives are predicted to incorporate better at high supersaturations, while the converse is expected for strongly-binding additives as there is less competition for their binding to the surface at low supersaturations.^45^

In the current work, we have shown that we can occlude particles that are an order of magnitude larger (≈2 μm) than any previously explored. This was achieved by functionalising the occlusion species with PAH, which is cationic under the reaction conditions. Our data also demonstrate that – due to the large size of the particles – the mechanism of incorporation within calcite single crystals is completely different to the processes responsible for the incorporation of small molecules and nanoparticles. Rather than binding to the crystal surface, the particles now act as “seeds” on which new crystals nucleate and then grow. In the vast majority of the systems explored, namely unmodified Ehux cells, and cells functionalized with PAA (anionic) and PEG (neutral) polymers, these crystallites grow to generate a polycrystalline shell. Only in the case of PAH were single crystals observed.

The mechanism by which this occurs is intriguing. Given that the calcite crystals that form on the PAH-functionalized coccospheres are oriented in multiple directions, their mutual continued growth will not lead to a single crystal product. A single crystal can therefore only develop if one crystal grows at the expense of the others, potentially *via* an Ostwald ripening process. That such a mechanism may operate is supported by the slow transformation, where perfect calcite single crystals are only observed after 4 days incubation in solution. Clearly, the PAH plays a significant role as single crystals were not observed in its absence. We postulate that the PAH offers judicious balance between promoting calcite nucleation on the surface of the PAH-functionalised particle, and retarding growth in solution, such that Ostwald ripening to a single large crystal can occur.

The incorporation of the micron-sized carrier particles within single crystals of calcite serves two main purposes: it prevents the leakage of the encapsulated active species to the external medium, and provides them with protection from environmental factors that can cause degradation, such as humidity, UV light and atmospheric oxygen. As calcite is biocompatible and dissolves readily at acidic pH values, it could serve for the oral delivery of different active pharmaceutical ingredients. That we can encapsulate both biological cells and synthetic hollow calcite spheres demonstrates the generality of our approach as a protection/delivery system, where commercially-available PAH can potentially be used to drive the occlusion of a wide range of additives that would not incorporate in its absence. This method can therefore be further optimized for targeted delivery applications, taking advantage of the host crystal lattice to provide occluded molecules with additional protection against environmental factors. In this respect, the use of synthetic hollow spheres of calcite would be preferable over the use of living organisms. The advantage of using micron-sized objects over nanoscale additives as carriers is that their internal volumes are several orders of magnitude larger (≈14 μm^3^) for a 3 μm-sized calcite hollow sphere, *versus* ≈ 0.0005 μm^3^ for a 100 nm micelle. Thus, they enable the loading of much larger volumes of active species into calcite.

## Conclusions

Building on previous work which has explored the occlusion of species ranging from organic molecules, to organic and inorganic nanoparticles, and sub-micron polymer particles within single crystals, we have here described a strategy that leads to the incorporation of micron-scale particles within calcite single crystals. Given the much larger volume occupied by these large particles within each calcite crystal as compared with the typical loadings of nanoparticles, this has great potential as an effective protection/delivery system for active compounds. Key to our approach was the functionalisation of the particles with poly(allylamine hydrochloride) (PAH) which is positively charged under the reaction conditions (pH 9). This strategy was demonstrated for two complementary particles – coccospheres and hollow calcium carbonate shells – and the encapsulation mechanism was shown to be completely distinct from that which leads to the occlusion of nanoparticles. Further work will address the mechanism by which PAH mediates the occlusion of micron-scale particles within the host crystal and will explore the use of these nanocomposites in delivery applications.

## Experimental

### Materials

All chemical reagents were purchased from Sigma-Aldrich and were used without further purification. Water used in the crystallization experiments was of HPLC grade and was purchased from Fisher Scientific. Seawater used to maintain algae cultures was provided by the Culture Collection of Algae and Protozoa (Scottish Association for Marine Science, Oban, United Kingdom).

### Methods

#### *Emilainia huxleyi* (‘Ehux’) cultures

Ehux (strain 982/1) culture was established using cells provided by the Culture Collection of Algae and Protozoa (Oban, UK). Parent Ehux cultures were used for subculturing once the concentration of cells stabilized at 3.5–106 cells per mL. The final composition of newly established culture media is presented in Table 1. The cells were incubated in an incubator (Panasonic MLR-352 Versatile Environmental Test Chamber) at 15 °C using the 12 h/12 h day/night cycle (photosynthetic photon flux density 200 μmol m^−2^ s^−1^).

**Table 1:** Composition of the medium used to culture Ehux cells

Ingredient	Aldrich cat. no	Volume [mL]
Seawater	—	28.1
Guillard’s (F/2) marine water enrichment solution (with silicate)	G9903	0.6
Antibiotics solution (penicillin–streptomycin–neomycin)	P4083	0.3
Parent culture	—	1.0
Total		30.0

#### Calcite crystal growth – general experimental procedure

Calcium carbonate was precipitated by mixing equimolar aqueous solutions of calcium chloride and sodium bicarbonate. Unless stated otherwise, the initial concentrations of Ca^2+^ and HCO_3_^−^ in the growth mixtures were adjusted to 20 mM. Crystallization was carried out in 10 mL glass vials equipped with *ca.* 3 mm × 5 mm glass substrates that had been cleaned with Piranha solution prior to calcite deposition.

#### Calcite containing *Emiliania huxleyi* (‘Ehux’) occlusions

When the concentration of Ehux cells in the growth medium reached 3 500 000 cells per mL, which was recorded after *ca.* 10 days since subculturing, 1 mL of the culture medium was placed in a centrifuge tube and was centrifuged at 2500 rpm for 10 minutes in order to separate the cells from seawater. The remaining liquid was decanted and the cells were re-dispersed in 1 mL of a 20 mM aqueous solution of CaCl_2_. 4 μL of 0.25% aqueous solution of poly(allylamine hydrochloride) (‘PAH’, *M*_w_ = 17.500 g mol^−1^, Sigma Aldrich cat. no. 283215) were added to the centrifuge tube and the mixture was gently shaken for about 1 minute. After that time, the Ehux containing PAH/CaCl_2_ mixture was placed on the surface of a Piranha-cleaned glass substrate located at the bottom of 10 mL glass vial and 1 mL of freshly prepared 20 mM NaHCO_3_ mixture was added to the Ehux/PAH/CaCl_2_ solution. In order to achieve complete occlusion of coccospheres within single crystalline calcite host crystals, calcite was allowed to grow for at least 96 hours. After that time, the glass substrates were removed from the vials, washed with ethanol, dried using a stream of air, and imaged using optical and electron microscopes.

Partially mineralized samples used in time-resolved SEM experiments were obtained following the same procedure. However, in that case, the crystallization time was varied between 5 minutes and 48 hours.

Practical remarks: The occlusion of Ehux coccospheres was efficient, with above 85% (optical microscopy estimation) of coccospheres present in the sample becoming incorporated within calcite single crystals. In order to maximize the occlusion efficiency, the following steps were taken:

Coccospheres were treated gently, especially during the centrifugation. Any increase of the centrifugation speed above 2500 rpm led to increased numbers of loose coccoliths present in the sample, suggesting that the mechanically interlocked coccospheres were not able to withstand the increased centrifugal force.

The usage of clean water was avoided – Ehux cells burst when exposed to clean water due to the increased osmotic pressure, destroying the coccospheres. According to our observations, the coccospheres were preserved for the time required to carry out their occlusion when Ehux cells were dispersed in 20 mM solution of CaCl_2_.

#### Using Ehux coccospheres as carriers for organic molecules

10 μL of 1 mM solution of Nile Red (Sigma Aldrich cat. no. 19123) in DMSO was added to 30 mL of Ehux culture. The cells were incubated in the presence of the fluorescent dye for 12 hours (night cycle). After that time, they were separated from the growth medium using centrifugation, re-dispersed in an aqueous 20 mM solution of CaCl_2_, and occluded within calcite single crystals using the procedure described above.

Practical remark: although the cells tolerated the presence of Nile Red (tested up to 10 μM) and DMSO (up to 1% v/v) in the medium, exposing Ehux cultures to these organic compounds for a longer time did not offer any substantial benefit. Also, we noted that when Nile Red was added to the Ehux culture medium during the day phase, it rapidly photodegraded to non-fluorescent derivatives.

#### Using Ehux coccospheres as carriers for inorganic nanoparticles

25 μL of 1% aqueous solution of CdTe quantum dots (*λ*_em_ = 570 nm) coated with carboxylate functionalities (Sigma Aldrich cat. no. 777943) were added to 30 mL of Ehux culture. The cells were allowed to grow in the presence of the quantum dots for 12 hours (during the night phase). After that time, they were separated from the CdTe-containing growth medium using centrifugation, re-dispersed in 20 mM aqueous solution of CaCl_2_ and occluded within single-crystalline calcite hosts using the method described above.

#### Synthesis of synthetic calcite hollow spheres (‘SCHS’)

SCHS were precipitated from water/hexane mixtures following a literature procedure reported by Fujiwara *et al.* Briefly: 32 mL of freshly prepared 3 M K_2_CO_3_ solution was rapidly mixed with 48 mL of hexane containing 0.67 g of Tween 80 and 0.33 g of Span 80. The obtained emulsion was then poured into 640 mL of 300 mM CaCl_2_ solution in water and stirred at 400 rpm for 10 minutes. After that time, the precipitated solid particles were filtered from the remaining emulsion using a 0.22 μm Millipore membrane, washed with an excess of 10 mM aqueous solution of CaCl_2_ and ethanol, and dried in an oven, at 110 °C. In order to precipitate SCHS pre-loaded with a fluorescent dye, 0.2 mg of 8-hydroxy-1,3,6-pyrenetrisulfonic acid (‘HPTS’) was added to the aqueous K_2_CO_3_ solution prior to emulsification.

#### Synthesis of poly(allylamine hydrochloride-*co*-(fluorescein)) (‘PAH-FL’)

Fluorescein-labelled poly(allylamine hydrochloride) was synthesised from 15 000 g mol^−1^ PAH (Sigma-Aldrich, cat. no. 283215) and fluorescein isothiocyanate (Sigma-Aldrich, cat. no. F4274) followed by recrystallisation and lyophilisation under vacuum, according to previously published procedures.^46^

#### Scanning electron microscopy (‘SEM’)

SEM imaging was performed using a Zeiss Evo scanning electron microscope equipped with a Gemini column and a field emission gun, operating at 3 kV. In order to investigate the dimensions and morphological details of the precipitated crystals, samples deposited on glass slides were fixed on aluminum stubs using double sided copper tape, coated with a thin layer (*ca*. 5 nm) of gold, and imaged using the secondary electron detector.

#### Confocal laser scanning microscopy

Crystals containing fluorescent occlusions were imaged using a Leica SP5 inverted confocal microscope equipped with a set of diode and gas lasers. Samples deposited on glass slides were coated with a thin layer of high refractive index oil and then were placed up-side down on a thin microscope cover glass. Focal plane merging acquisitions (*z*-stacks) were recorded at 4096 × 4096 resolution by averaging three frames recorded at the same focal length. Image analysis was carried out using the FiJi software package.

#### Raman microscopy

Raman spectra were acquired using the Renishaw inVia confocal Raman microscope equipped with a 785 nm IR laser. Spectra were recorded in the 1200 cm^−1^ to 100 cm^−1^ range and the total acquisition time was 60 seconds per spectrum.

## Conflicts of interest

There are no conflicts to declare.

## Supplementary Material
